# Global Heated Tobacco Product User Estimates, 2014-2024: Descriptive Surveillance Study Using Manufacturer Disclosures

**DOI:** 10.2196/88761

**Published:** 2026-04-20

**Authors:** Giorgi Mzhavanadze, Gerry V Stimson, Tomasz Jerzyński

**Affiliations:** 1 Knowledge-Action-Change, 5 Heatherfold Way, London, HA5 2LG, United Kingdom, 44 01708 226231; 2 Healthy Initiatives (GO Zdorovi Iniciativi), Kyiv, Ukraine; 3 Center for Social Research Methodology, Robert Zajonc Institute for Social Studies (ISS), University of Warsaw, Warsaw, Poland

**Keywords:** heated tobacco products, global estimates, tobacco and nicotine market, tobacco industry data, user prevalence, surveillance and monitoring

## Abstract

**Background:**

Heated tobacco products (HTPs) expanded rapidly after 2014; yet globally, comparable estimates of the number of HTP users remain limited. National surveys rarely include standardized HTP measures, and international surveillance systems do not provide harmonized global user counts.

**Objective:**

The aim of the study is to construct a reproducible annual global time series of HTP user estimates for 2014‐2024 using publicly available manufacturer disclosures, quantify uncertainty, and compare these estimates with user counts derived from nationally representative survey prevalence data where available.

**Methods:**

We compiled annual HTP user counts and heated-tobacco stick shipment volumes from publicly available reports of major transnational tobacco companies. In the primary series, we used company-reported user counts when available. For company-year observations without reported user counts, we estimated user numbers by converting reported shipment volumes into implied user counts, applying brand-specific assumptions on average daily consumption per user. As an alternative sensitivity analysis, all company user counts were excluded, and all shipments were converted using a common literature-based consumption parameter. As a complementary check, nationally representative current-use prevalence data from 35 countries were converted to user counts.

**Results:**

Global HTP users reached 48.9 million in 2024 in the primary series (range 45.6‐52.1 million). The shipment-only series showed higher totals (67.9 million; range 59.7‐78.7 million). Survey-based estimates yielded 21.8 million users across 35 countries, reflecting partial geographic coverage.

**Conclusions:**

A transparent, updateable global time series documents rapid HTP growth from 2014 to 2024, with 2024 totals ranging from 45.6 to 78.7 million users depending on the estimation approach. As standardized HTP measures are incorporated into national surveys, future estimates can be refined, and uncertainty reduced.

## Introduction

Heated tobacco products (HTPs) are electronic devices designed to heat processed tobacco and generate an aerosol without burning the tobacco. Unlike conventional cigarettes, which combust tobacco at high temperatures (>600 °C), HTPs typically operate at lower temperatures (250‐350 °C) using an electrically powered heating element. HTP systems generally consist of a specially manufactured tobacco consumable (commonly in the form of sticks), a heating mechanism, and a battery-powered control unit that regulates device operation [1-3].

Introduced commercially in 2014, HTPs have become a notable segment of the global nicotine market, expanding rapidly from initial launches in Japan and South Korea [4-9]. Despite this expansion, globally comparable estimates of the number of HTP users remain scarce. National tobacco surveys rarely include HTP questions, and where they do, definitions and measurement approaches vary, limiting cross-country comparability and time-series analysis. The most comprehensive systematic reviews to date highlight persistent surveillance gaps and heterogeneity in measures [4,5]. These reviews reflect prevalence data from surveys conducted up to 2020 and identify current adult HTP prevalence estimates from 34 countries; only a small number of countries (Japan, Italy, the United States, and the United Kingdom) contribute 3 or more prevalence estimates across different years [5]. By contrast, HTPs are legally available in at least 72 countries as of 2025 [10], indicating a substantial mismatch between product availability and the coverage of nationally representative prevalence evidence. To date, global surveillance has not produced an annual time series of the number of HTP users that spans the full period of commercial uptake.

Similar measurement challenges previously existed for other noncombustible nicotine products, e-cigarettes, where global user estimates were initially constructed by compiling heterogeneous sources and then iteratively updated as evidence improved [11-13]. The World Health Organization has recently reported adult e-cigarette user estimates based on nationally representative surveys. However, a comparable evidence base does not yet exist for HTP, which are not consistently separated from broader smoked tobacco categories [14]. A systematic assessment of Eurobarometer tobacco surveys documented that HTP-specific questions were not introduced until 2020 (6 years after HTPs entered European markets) and identified significant inconsistencies in question wording and product definitions across survey waves, further limiting comparisons [15].

This study addresses that empirical gap by generating a global time series of HTP user estimates for 2014‐2024. In contrast with existing estimates, which are primarily survey-based, we systematically compile manufacturer-reported user counts and sales volumes and convert volumes to user numbers when necessary using explicit assumptions about daily consumption. The rationale for using company data is pragmatic: such disclosures are often the only publicly available and regularly updated source of information on global HTP uptake. Furthermore, the HTP market is highly concentrated among a small number of multinational firms [16,17], which allows the construction of consistent and comprehensive annual estimates.

The aim of this study is to construct a transparent and reproducible global time series estimate of the number of HTP users from 2014 to 2024 using publicly available manufacturer disclosures. Specifically, we address the following research questions: (1) What is the estimated global number of HTP users annually from 2014 to 2024 based on manufacturer-reported data? (2) How do estimates differ across alternative estimation approaches: company-reported user counts, shipment-based conversions, and survey-based prevalence? (3) What are the principal sources of uncertainty in these estimates, and how sensitive are results to key methodological assumptions?

## Methods

### Overview

**Figure 1.**
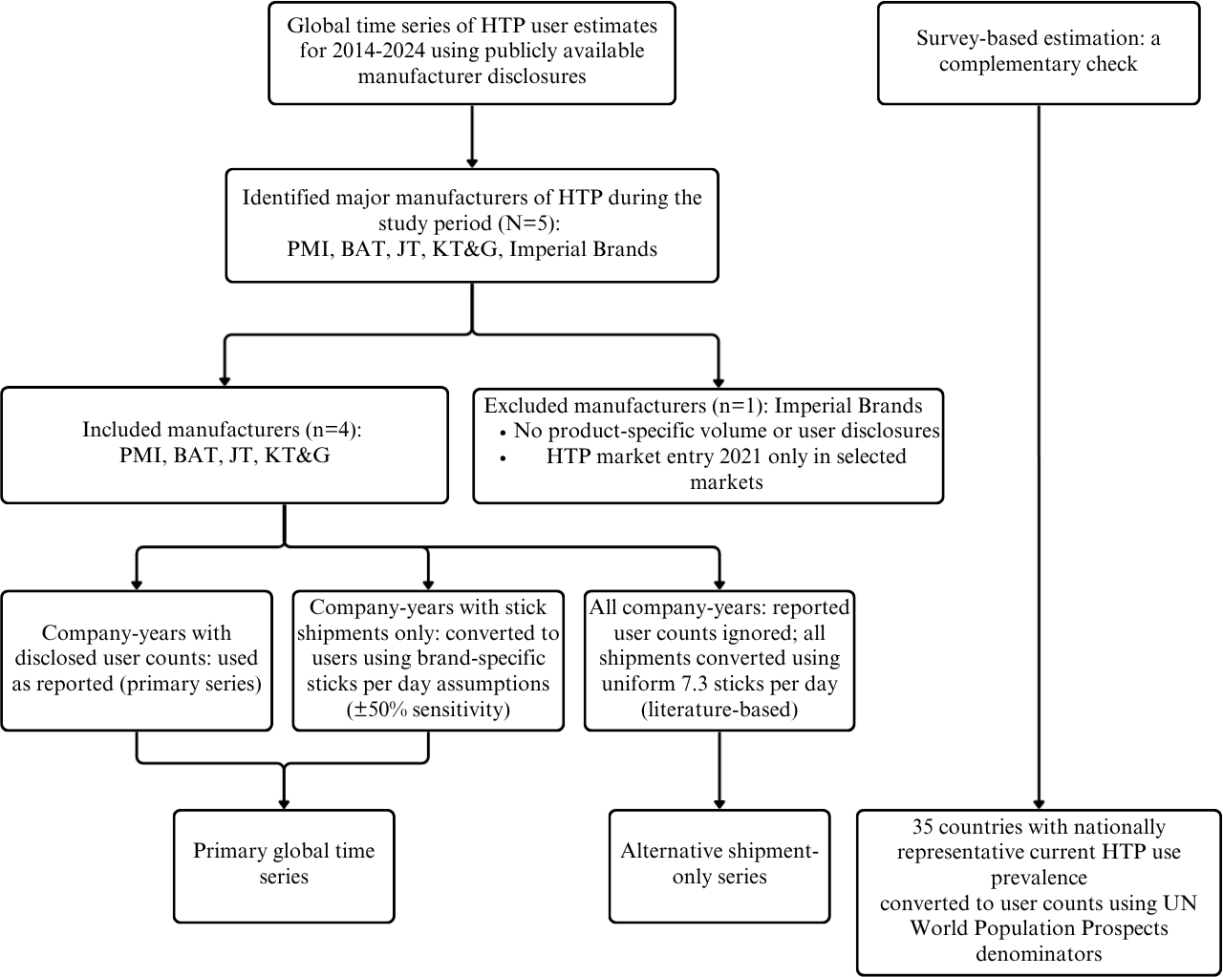
Data identification and estimation workflow for global HTP user estimates, 2014‐2024. BAT: British American Tobacco; HTP: heated tobacco product; JT: Japan Tobacco; KT&G: Korea Tobacco & Ginseng Corporation; PMI: Philip Morris International.

This section describes the study design, data sources, analytical procedures, and ethical considerations. The study is a descriptive global surveillance analysis based on secondary data from publicly available manufacturer disclosures. No primary data were collected. Figure 1 summarizes the data identification and estimation workflow.

### Study Design

This study estimates the global number of HTP users from 2014 to 2024 by synthesizing secondary data from publicly available manufacturer disclosures to construct a replicable annual time series. Two parallel disclosure-based global series were developed to address definitional heterogeneity and reporting differences across manufacturers: a primary series combining company-reported user counts with shipment-based conversions, and an alternative series relying solely on shipment-based conversions using a common literature-based consumption parameter. As a complementary check, nationally representative survey prevalence data were converted to user counts independently.

### Data

Manufacturer-reported data were extracted from publicly available corporate disclosures (annual reports, investor presentations, regulatory filings, and press releases). These sources provided information on (1) HTP user or consumer counts and (2) annual shipment volumes of HTP consumables (sticks), typically reported at the global level [18-22].

Companies were included if they (1) manufactured and sold HTPs at a material global scale during 2014‐2024 and (2) publicly disclosed annual user counts or stick shipment volumes sufficient to construct year-by-year estimates. Five major manufacturers of HTP were identified during the study period: Philip Morris International (PMI; IQOS), British American Tobacco (BAT; glo), Japan Tobacco (JT; Ploom), Korea Tobacco & Ginseng Corporation (KT&G; lil), and Imperial Brands. Four companies met the inclusion criteria: PMI, BAT, JT, and KT&G. Imperial Brands was excluded because the company entered the HTP market only in 2021 in selected European markets and does not disclose product-specific volumes or user counts [23]. No other manufacturers met the inclusion threshold of material global scale and public disclosure. For each included company, all available annual observations from 2014 to 2024 were extracted. Where multiple disclosures existed for a single company-year, the most recent published figure was used.

Reporting conventions differed across companies. PMI and BAT publish headline user or consumer counts alongside annual HTP stick shipments. PMI reports “total IQOS users,” defined as adults who used PMI HTPs for at least part of their daily tobacco consumption in the past 7 days, and in some reports, also distinguishes “switched” users. BAT reports user counts only in selected years and does not provide a formal definition. JT does not disclose global user counts; instead, it reports stick volumes in selected years and, more recently, market-share indicators based on stick volumes. KT&G generally reports stick shipments and does not publish global user counts.

Notably, under the PMI-KT&G distribution or supply agreement, initiated in January 2020 (extended in January 2023), PMI commercializes KT&G’s HTPs outside South Korea [24]. PMI’s disclosures state that, from December 2020, its reported figures include licensed KT&G products in non-Korean markets [25]. To prevent double-counting, we therefore treat PMI’s 2021 onward published user totals as already inclusive of KT&G users outside South Korea. KT&G’s domestic (South Korea) stick shipments are converted to user estimates and counted separately, while KT&G volumes outside South Korea are not added on top of PMI’s user totals.

Table 1 summarizes the availability of company-reported user counts and stick shipment data by company and year. It documents the availability and structure of input data rather than estimated results and functions as a data inventory.

**Table 1.** Manufacturer-reported heated tobacco product (HTP) user counts and heated-tobacco stick shipment volumes used to construct global HTP user estimates, by company and year, 2014‐2024^a^.

Year	PMI^b^ (IQOS)		KT&G^c^ (lil)		BAT^d^ (glo)		JT^e^ (Ploom)	
Users (million)	HTP sticks (billion)	Users (million)	HTP sticks (billion)	Users (million)	HTP sticks (billion)	Users (million)	HTP sticks (billion)
2014	N/A^f^	N/A	0.0	0.0	0.0	0.0	0.0	0.0
2015	N/A	N/A	0.0	0.0	0.0	0.0	0.0	0.0
2016	2.1	7.4	0.0	0.0	N/A	N/A	0.0	0.0
2017	6.9	36.2	N/A	0.0	N/A	2.2	0.0	0.0
2018	9.6	41.4	N/A	1.2	N/A	7.0	0.0	0.0
2019	13.6	59.7	N/A	2.5	3.0	9.0	N/A	N/A
2020	17.6	76.1	N/A	1.5	4.0	11.0	N/A	N/A
2021	21.2	95.0	N/A	3.7	6.8	19.0	N/A	3.0
2022	24.9	109.2	N/A	5.8	7.2	24.0	N/A	6.7
2023	28.6	125.3	N/A	8.2	8.6	24.0	N/A	11.1
2024	32.2	139.7	N/A	8.3	10.2	21.0	N/A	14.1

a IQOS, lil, glo, and Ploom are heated tobacco brand names.

b PMI: Philip Morris International.

c KT&G: Korea Tobacco & Ginseng Corporation.

d BAT: British American Tobacco.

e JT: Japan Tobacco

f N/A: not available.

### Analysis

To address definitional heterogeneity and variable disclosure completeness across manufacturers, we constructed 2 parallel global time series.

#### Primary Time Series: Company-Reported Users and Shipment-Based Conversions

For company-years with disclosed HTP user counts, we used those values as reported. For company-years with only HTP stick shipment volumes, we converted shipments to implied user counts using:


$${\text{Users}}_{y}\text{=}\frac{{\text{HTP stick shipments}}_{y}}{\text{average number of sticks}\text{ per user per day}\times 365}$$

Brand-specific assumptions were applied as follows: (1) KT&G—12 sticks per day (derived from years in which PMI disclosed both sticks and users) and (2) BAT and JT—7.6 sticks per day (based on BAT years reporting both sticks and users). For each year, we then aggregated company-level user counts (reported or converted) across firms to produce global totals from 2014 to 2024.

Additional assumptions were required where data were unavailable:

- JT’s global heated tobacco stick share in 2017‐2018 is assumed to be equal to its 2019 level (2.5%).
- BAT launch year: glo launched in December 2016; we assume 0 consumers for 2016 [26].
- BAT revisions: When BAT revises historical “consumer acquisition” figures in later reports, we use the most recent published figures for that year.
- PMI launch year: IQOS launched in 2014; given that the national prevalence of HTP use in Japan was 0.2% in 2015, we set 0 users in 2014 [27].
- KT&G 2020 market split: The domestic versus international split for KT&G in 2020 is not reported. We assume the proportion matched the 2021 level.

Because daily stick consumption might vary by brand, device generation, market maturity, and user profile, we applied a ±50% sensitivity around each brand’s central sticks-per-day assumption when converting stick volumes to users. Lower and upper bounds were reported alongside central values for any company-year derived from shipments. No sensitivity was applied to directly reported user counts.

#### Alternative Time Series: Shipment-Only Conversions Using Literature-Based Consumption

To address uncertainty arising from inconsistent or noncomparable manufacturer user definitions, we constructed an alternative series that ignores all manufacturer-reported user counts and converts all companies’ stick shipments to users using a common consumption parameter derived from the literature.

External survey evidence from Germany (2018‐2023) indicates that current HTP users reported a mean consumption of 7.3 sticks per day, with IQOS accounting for 84% of current use [28]. Comparable estimates were observed in Italy (2018‐2020), where IQOS users reported a mean consumption of 7.9 sticks per day [29].

Based on these findings, we applied a central estimate of 7.3 sticks per day and used the corresponding 95% CI (6.33‐8.27 sticks per day) to generate lower and upper bound user estimates, respectively.

#### Estimation Using National Survey Prevalence

To complement the industry disclosure-based series, we developed an independent survey-based estimate of HTP users derived from nationally representative prevalence data where available. We extracted country-level estimates of current HTP use from 35 countries compiled in the Global State of Tobacco Harm Reduction database, which combines data from Eurobarometer 2023, Global Adult Tobacco Surveys conducted between 2019 and 2023, World Health Organization’s STEPwise approach to noncommunicable diseases risk factor surveillance surveys conducted between 2019 and 2022, and recent nationally representative surveys [10].

For each country, the reported prevalence of current HTP use was multiplied by the corresponding adult population for the survey year, aligned with the survey’s defined age range (eg, 15+, 18+, 16‐74, or 19+ years), using population denominators from the United Nations World Population Prospects [30]. Country-specific user counts were then aggregated to produce a combined total across the 35 countries. All data sources used for this estimation are presented in Table 2.

**Table 2.** Nationally representative survey inputs used to estimate heated tobacco product (HTP) users in 35 countries.

Country	Current HTP use prevalence	Year	Survey	Cohort	Population (thousands), n	Estimated HTP users, n
Austria	3.00	2023	Eurobarometer	15+	7764.2	232,927
Belarus	3.00	2020	STEPS^a^	18+	7571.0	227,131
Belgium	1.00	2023	Eurobarometer	15+	9717.5	97,175
Bulgaria	2.00	2023	Eurobarometer	15+	5833.4	116,669
Croatia	2.00	2023	Eurobarometer	15+	3348.3	66,966
Cyprus	5.00	2023	Eurobarometer	15+	1117.9	55,895
Czechia	4.00	2023	Eurobarometer	15+	8971.1	358,843
Estonia	2.00	2023	Eurobarometer	15+	1130.9	22,619
Georgia	1.60	2021	Local	18+	2873.7	45,980
Germany	1.00	2023	Eurobarometer	15+	72,387.7	723,877
Greece	3.00	2023	Eurobarometer	15+	8970.5	269,116
Hungary	3.00	2023	Eurobarometer	15+	8275.2	248,255
Ireland	1.00	2023	Eurobarometer	15+	4115.8	41,158
Italy	4.00	2023	Eurobarometer	15+	52,232.4	2,089,297
Japan	11.80	2022	Local	16‐74	90,240.2	10,648,339
Jordan	4.10	2025	Local	15+	7670.8	314,503
Latvia	3.00	2023	Eurobarometer	15+	1581.5	47,445
Lithuania	5.00	2023	Eurobarometer	15+	2393.0	119,650
Luxembourg	1.00	2023	Eurobarometer	15+	549.6	5496
Netherlands	1.00	2023	Eurobarometer	15+	15,155.9	151,559
Philippines	0.10	2021	GATS^b^	15+	77,692.2	77,692
Poland	1.00	2023	Eurobarometer	15+	32,503.6	325,036
Portugal	5.00	2023	Eurobarometer	15+	9062.6	453,131
Romania	2.00	2023	Eurobarometer	15+	16,097.0	321,941
Slovakia	4.00	2023	Eurobarometer	15+	4599.2	183,969
Slovenia	2.00	2023	Eurobarometer	15+	1795.9	35,918
South Korea	5.90	2022	Local	19+	44,374.2	2,618,081
Spain	1.00	2023	Eurobarometer	15+	41,325.7	413,257
Mexico	0.20	2023	GATS	15+	96,059.4	192,119
Costa Rica	0.04	2022	GATS	15+	4033.0	1613
Ukraine	3.00	2023	Local	18+	33,723.9	1,011,717
Moldova	2.60	2021	STEPS	18+	2354.7	61,223
Kazakhstan	1.00	2019	GATS	15+	13,478.2	134,782
Armenia	0.80	2022	Local	15+	2294.9	18,359
Uzbekistan	0.24	2019	STEPS	18+	21,492.9	50,508

a STEPS: World Health Organization STEPwise approach to noncommunicable diseases risk factor surveillance.

b GATS: Global Adult Tobacco Survey.

### Ethical Considerations

This study used only publicly available, aggregated secondary data and did not involve identifiable personal information. Therefore, it does not meet the definition of human subjects research under applicable regulatory frameworks, and formal ethics committee approval was not required. This determination is consistent with the US Common Rule (45 CFR 46.102) [31], which defines human subjects research as involving identifiable private information or interaction with individuals, and with the European Union General Data Protection Regulation (Regulation [EU] 2016/679) [32], which applies only to personal data.

## Results

### Overview

This section presents the findings from the 3 estimation approaches. We first report the primary disclosure-based time series, then the alternative shipment-only series, and finally the survey-based estimation. Results are organized to address the 3 research questions outlined in the Introduction section.

The estimation drew on 44 possible company-year observations (4 companies×11 years, 2014‐2024). User counts were directly reported by manufacturers for 25 (56.8%) of these observations, while stick shipment volumes were available for an additional 14 (31.8%) observations, resulting in 39 (88.6%) company-year observations with at least 1 usable data point. For the remaining 5 (11.4%) observations, primarily early years before product launch or before public disclosure began, values were set to 0 or imputed using the documented assumptions described in the Methods section. No company-year observations were excluded from the analysis due to missing data; all gaps were addressed through the documented imputation and conversion procedures.

### Primary Time Series: Company-Reported Users and Shipment-Based Conversions

**Figure 2.**
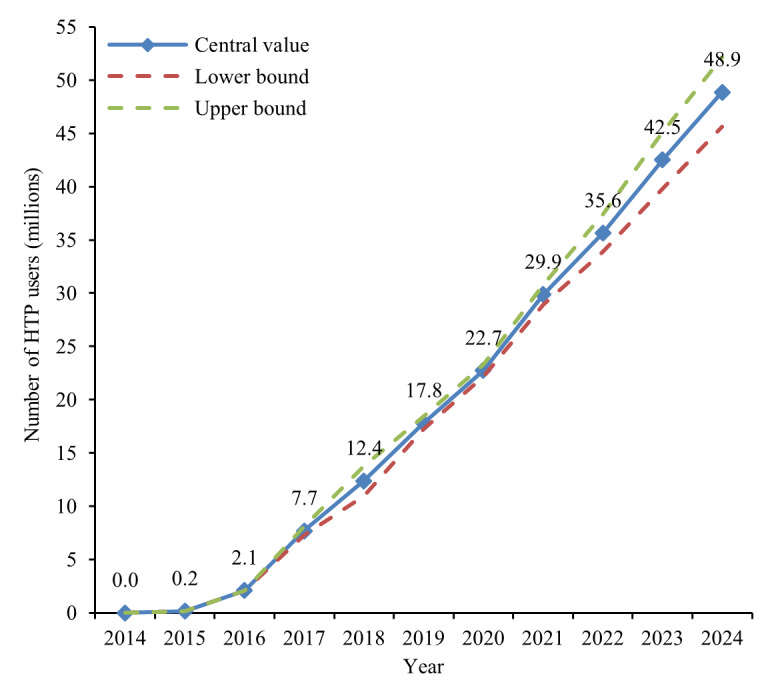
Global number of HTP users, 2014‐2024—primary disclosure-based time series. Dashed lines indicate lower and upper bounds based on ±50% sensitivity on daily consumption assumptions. HTP: heated tobacco product.

The number of HTP users grew from negligible levels in 2014 to an estimated 48.9 million in 2024 (Figure 2). The estimates indicate a consistent upward trend across the decade, with the lower and upper bounds for 2024 ranging from 45.6 to 52.1 million, respectively, reflecting the ±50% sensitivity applied to shipment-based conversion assumptions. Because more than half of the 2024 global total is anchored in directly reported user counts, the overall uncertainty range remains relatively narrow.

**Figure 3.**
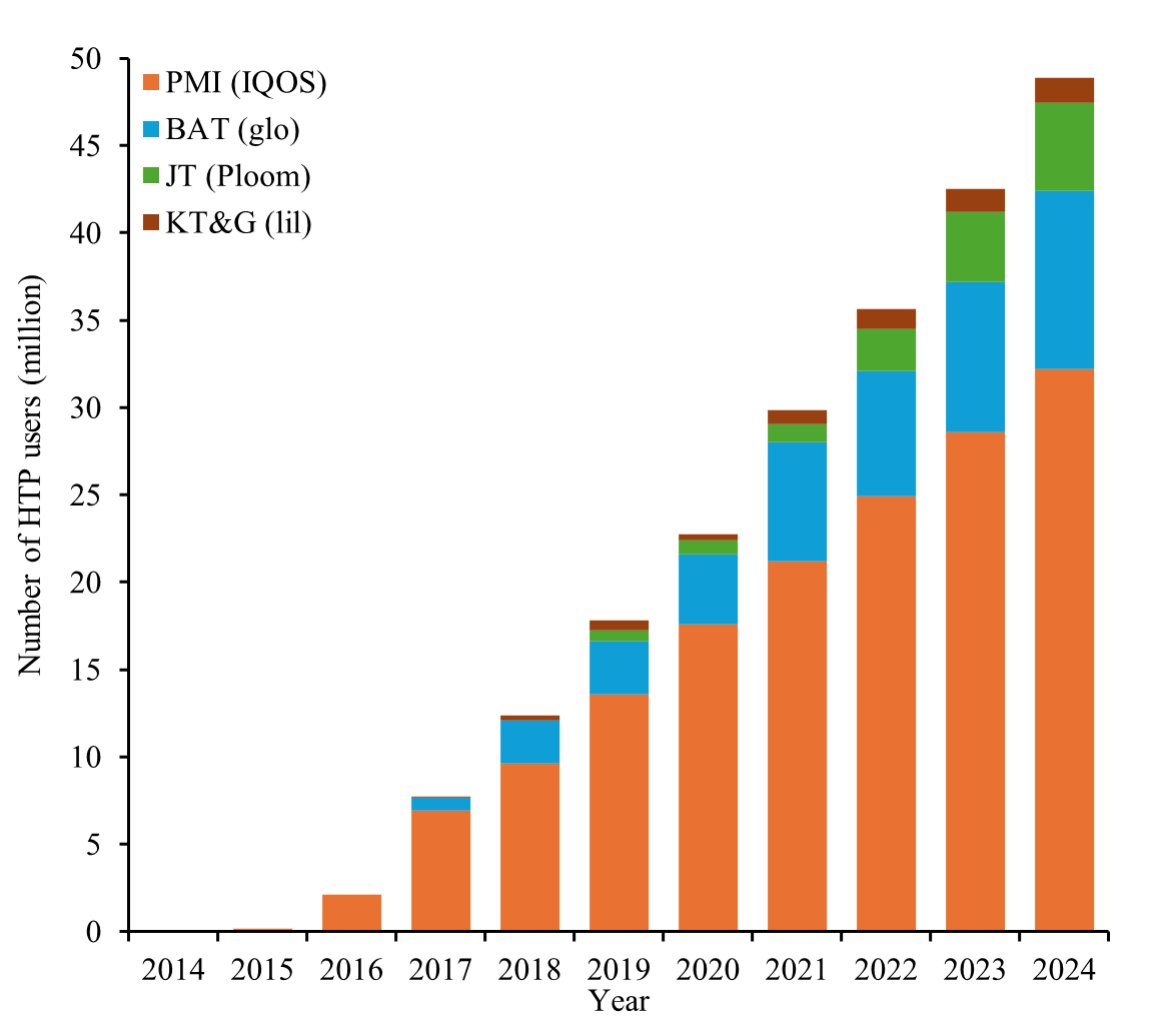
Estimated global HTP users by manufacturer, 2014‐2024—primary disclosure-based series. IQOS, lil, glo, and Ploom are heated tobacco brand names. BAT: British American Tobacco; HTP: heated tobacco product; JT: Japan Tobacco; KT&G: Korea Tobacco & Ginseng Corporation; PMI: Philip Morris International.

Growth was driven primarily by PMI’s IQOS, which accounted for the majority of users throughout the period (Figure 3). BAT’s glo gained traction from 2017 onward, while JT’s Ploom contributed smaller but steadily increasing shares. KT&G’s lil maintained a modest share.

### Alternative Time Series: Shipment-Only Conversions Using Literature-Based Consumption

**Figure 4.**
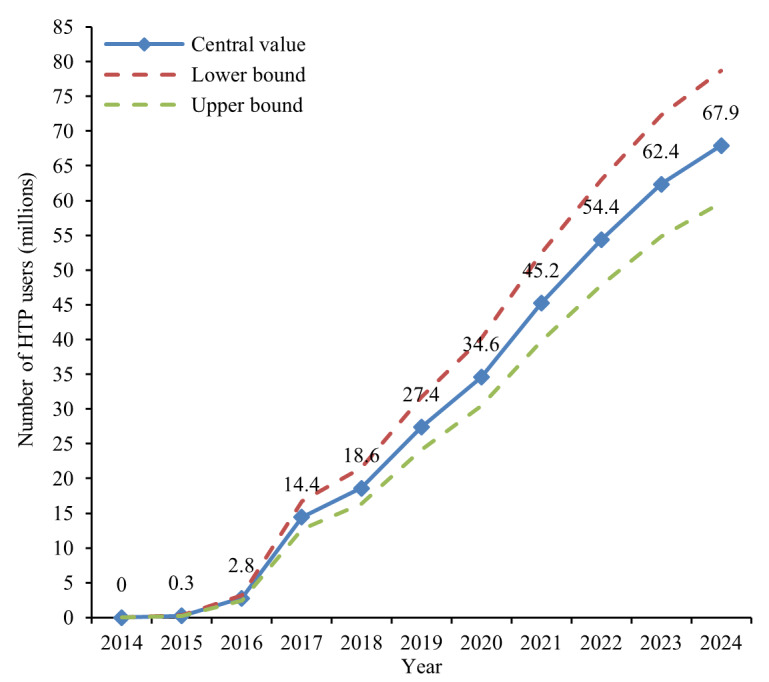
Global number of HTP users, 2014‐2024—alternative shipment-only time series using literature-based consumption assumptions. Alternative series converting all manufacturer stick shipments to user estimates using a uniform 7.3 sticks per day assumption (95% CI 6.33‐8.27). Dashed lines indicate lower and upper bounds. HTP: heated tobacco product.

Applying a uniform 7.3 (95% CI 6.33‐8.27) sticks per day, consumption assumption to all firms’ shipment volumes yields a consistently higher series, reflecting the more conservative daily consumption assumption and the removal of unclear company user definitions (Figure 4). In 2024, this approach produces an estimated 67.9 million users (range 59.7‐78.7 million). The wider uncertainty interval compared with the primary series is expected, because all company-years are derived from shipment conversions, and none are anchored by directly reported user counts. Despite higher absolute levels, the trajectory closely mirrors the primary series.

### Estimation Using National Survey Prevalence

Nationally representative prevalence data yielded an estimated 21.8 million HTP users across 35 countries (Table 2). The largest numbers were observed in Japan (10.6 million in 2023), South Korea (2.6 million in 2022), Italy (2.1 million in 2023), Ukraine (1.0 million in 2023), and Germany (0.7 million in 2023). These 5 countries accounted for 78.5% (17.1 million) of the survey-based total. Most available surveys were conducted in 2022 or 2023, with some dating to 2019‐2021 and only 1 in 2025, meaning that they may not capture more recent market expansion. The survey-based total of 21.8 million users across 35 countries is substantially lower than the disclosure-based estimates for the same period. Given partial geographic coverage, this should be interpreted as a conservative lower bound, rather than an alternative estimate of the global total.

## Discussion

### Principal Findings

This study addressed 3 research questions. First, using publicly available manufacturer disclosures combined with explicit shipment-to-user conversions where required, we constructed an annual global time series of HTP user estimates for 2014‐2024. The primary series indicates that global HTP users increased from negligible levels to 48.9 million in 2024 (range 45.6‐52.1 million).

Second, when all shipment volumes were converted using a standardized literature-based consumption parameter, the shipment-only sensitivity series yielded higher 2024 totals (67.9 million; range 59.7‐78.7 million) but preserved a similar growth trajectory. This upward shift is expected because the shipment-only approach imposes a uniform consumption conversion factor and removes manufacturer-specific user definitions, some of which may reflect partial use, different reference periods, or varying inclusion rules.

Across both disclosure-based series, the direction and magnitude of growth since 2014 were consistent: near 0 user numbers in early launch years, accelerated expansion after 2016, and sustained increases through 2024. While absolute totals varied depending on conversion assumptions and reporting definitions, the upward trajectory was robust across estimation strategies.

Third, survey-derived user counts based on nationally representative prevalence data in 35 countries yielded a combined total of 21.8 million users. This estimate should be interpreted as a conservative lower bound rather than a competing global total. This estimate covers fewer than half of the countries where HTPs are legally available and relies on survey years that are mostly 2023 or earlier, before full expansion in some markets. Moreover, prevalence surveys vary in age frames, question wording, and whether they capture “current use” in a manner comparable across countries. These limitations restrict the survey series’ ability to represent the full global market, but the estimates nonetheless provide an important external check for the disclosure-based totals and illustrate how incomplete surveillance coverage remains despite the rapid market expansion documented here.

These findings extend the existing literature on HTP adoption and surveillance. Prior work has primarily focused on country-specific prevalence, particularly in early-adopting markets such as Japan and South Korea, documenting rapid substitution dynamics and substantial category growth within a relatively short period [4-9,28,29,33]. Sun et al [4] synthesized 45 studies across 42 countries but identified adult current-use prevalence data for only 12 countries. More recently, Scala et al [5] conducted the most comprehensive systematic review to date, covering 76 studies published between 2015 and February 2022 and reporting current adult HTP prevalence from 34 countries based on surveys conducted prior to 2021.

Our survey-based analysis covers 35 countries and incorporates prevalence data from surveys conducted in years including 2025. The results align with prior reviews, which have documented that HTP use remains concentrated in certain markets. However, this approach demonstrates the structural limitation of prevalence-based aggregation when nationally representative data are available for only a subset of countries where HTPs are legally marketed.

Unlike prior syntheses based exclusively on survey data, the disclosure-based series presented here generates global totals that are not constrained by survey coverage, thereby addressing a key measurement gap in global HTP surveillance. Methodological precedent for combining survey and commercial data sources exists in the broader tobacco surveillance literature. A comparison of self-reported cigarette consumption from a national survey in England with retail sales data for 2011‐2018 showed closely aligned trends, supporting the validity of combining survey-based and sales-based approaches to monitor population-level tobacco product dynamics [34].

As of 2025, HTPs are legally available in at least 72 countries, with uptake concentrated in early-adopting and predominantly higher-income markets [10]. Consequently, global estimates must be interpreted in this context. Although tens of millions of HTP users remain small relative to the 1 billion global population of people who smoke cigarettes [35], HTPs can capture substantial shares of the tobacco market within countries where they are widely available. For example, HTPs represented 48.5% of PMI’s combined cigarette and HTP market in Japan, 31.8% in Hungary, 28.7% in Lithuania, and 23.9% in Greece, according to company disclosures from the third quarter of 2025 [36].

From a public health perspective, the implications of HTP growth depend on behavioral transitions rather than uptake alone. A decision-theoretic framework indicates that population-level benefits are most plausible when HTP uptake substitutes for cigarette smoking among adults who would otherwise continue to smoke, whereas dual use or uptake among never-smokers may reduce or negate potential benefits [37]. The evidence base summarized in recent reviews remains mixed and is shaped by heterogeneity in study designs and regulatory contexts. The Cochrane review concluded that evidence for HTP as cessation tools remains limited relative to other interventions and highlighted substantial uncertainty [38]. Scala et al [5] emphasized variability in dual use and switching patterns, indicating that aggregate uptake alone does not resolve whether an increase in HTP use represents displacement of cigarette smoking or supplementation. Evidence from Japan suggests partial displacement of cigarette sales after HTP introduction, while also showing heterogeneity in exclusive use versus dual use across survey designs and population subgroups [7,8,27,33]. These findings reinforce that the key policy question is not simply how many people use HTP, but how HTP uptake changes cigarette smoking trajectories over time.

### Strengths

This study has several strengths. First, it relies exclusively on publicly verifiable data sources and documents extraction, harmonization, and revision procedures, enhancing transparency and reproducibility. Second, the dual-series approach (combining a primary disclosure-based series with an alternative shipment-only sensitivity) allows readers to assess how estimation choices affect results and provides bounded rather than single-point estimates. Third, all data sources, assumptions, and conversion formulas are fully documented, enabling independent replication and updating as new disclosures become available. Finally, the analysis covers the full commercially relevant period for HTP (2014‐2024) and all manufacturers operating at a material global scale, providing comprehensive market coverage.

### Limitations

This study has several limitations that should be considered when interpreting the findings. First, the estimates presented here are derived from secondary data sources rather than from a single harmonized global survey. Manufacturer-reported user counts and shipment volumes were used because nationally representative HTP prevalence data remain unavailable for most countries. Although these disclosures are among the few consistently updated global sources, they may differ in definitions, reporting granularity, and geographic aggregation across firms and over time. Listed firms face strong incentives for accuracy: disclosures are governed by securities laws, audited or reviewed through established internal controls, and subject to penalties for material misstatement, while reputational and investor-relations risks further discourage misreporting. However, these safeguards do not eliminate error or definitional ambiguity, and they reduce the likelihood of systematic overstatement.

Second, where shipment volumes were converted into user estimates, the analysis relied on explicit assumptions regarding average daily consumption of HTP sticks per user. While these assumptions were transparently documented and subjected to sensitivity analysis, true consumption patterns may vary across countries, over time, and by user characteristics. As a result, the estimates should be interpreted as approximations rather than precise counts.

Third, the presence of illicit or gray-market distribution may affect the allocation of HTP use at the country level, particularly in jurisdictions where HTP sales are restricted or prohibited. However, HTPs are manufactured by a small number of multinational firms, and HTP consumables are technologically standardized and brand-locked, creating substantial barriers to informal or counterfeit production [16]. Consequently, most HTP sticks consumed globally are produced by these firms and are captured in shipment-based disclosures at the point of manufacture and are therefore included in the estimates presented here.

Fourth, the study does not distinguish between exclusive HTP use and dual use with cigarettes. Manufacturer disclosures and most national surveys do not provide information to allow consistent differentiation of these patterns. As a result, the estimates quantify the number of people using HTP but do not directly inform whether HTP uptake represents substitution for cigarette smoking or supplementation alongside continued smoking.

### Scope

The scope of this study is the global population of HTP users as estimated from manufacturer disclosures and a subset of national surveys. The estimates apply to the period 2014‐2024 and to the 4 major HTP manufacturers. They are most informative for understanding the scale and growth of the HTP category in a global market characterized by concentration among a small number of multinational manufacturers. They are less informative for geographic allocation, intensity of use distributions, or product-specific switching dynamics. Generalizability to country-level prevalence trends is therefore limited and depends on the extent to which manufacturer-reported volumes reflect geographically diverse consumption patterns.

The survey-based series is limited to 35 countries with available nationally representative prevalence data, representing fewer than half of the countries where HTPs are legally sold.

### Recommendations for Future Research

Several directions for future research emerge from this analysis. First, as national surveys increasingly incorporate standardized HTP measures, researchers should validate disclosure-based estimates against survey-derived counts at the country level to calibrate conversion assumptions and assess the reliability of manufacturer-reported user definitions. Second, future studies should link HTP uptake estimates with longitudinal data on behavioral transitions (switching, dual use, relapse, and initiation), as aggregate uptake alone does not resolve the key policy question of whether HTP growth displaces or supplements cigarette smoking. Third, the estimation framework introduced here could be extended to incorporate subnational data and to distinguish exclusive HTP use from concurrent cigarette smoking where data permit. Fourth, as additional consumption data become available from diverse markets, the brand-specific and literature-based sticks-per-day assumptions used in this study should be updated and validated against observed use patterns in a wider range of regulatory and cultural contexts.

### Significance

This study contributes to the tobacco and nicotine surveillance literature by providing a reproducible global time series of HTP users, offering population estimates that are currently missing from the evidence base and that are required for epidemiologic modeling, burden-of-disease estimation, and regulatory and fiscal evaluation. The transparent methodology and publicly available data sources enable regular updating as new disclosures become available, supporting ongoing monitoring of the evolving global nicotine product landscape. By generating bounded estimates across multiple approaches, this work provides a more complete empirical foundation for interpreting shifts in nicotine consumption patterns and for informing evidence-based tobacco policy at the global level. Overall, this work complements, rather than replaces, survey-based epidemiology and helps bridge the measurement gap until national surveillance systems expand and standardize HTP measures sufficiently to produce harmonized global estimates.

### Conclusions

This study provides a transparent and replicable global time series estimate of HTP use from 2014 to 2024, addressing a notable gap in international tobacco and nicotine surveillance. Depending on the estimation approach, global HTP user totals in 2024 range from 45.6 to 78.7 million users in the disclosure-based series, with survey-based estimates across 35 countries providing a conservative lower bound of 21.8 million. Across all approaches, the direction and timing of growth are consistent, indicating rapid and sustained expansion of HTP use over the past decade. The analysis documents the scale and trajectory of this growth and provides essential population denominators for interpreting market transitions, supporting epidemiologic modeling, and informing regulatory and fiscal policy.

## Abbreviations

BAT: British American Tobacco
HTP: heated tobacco product
JT: Japan Tobacco
KT&G: Korea Tobacco & Ginseng Corporation
PMI: Philip Morris International
